# Feasibility of a combined online and in-person training model on Infant and Young Child Feeding (IYCF) counselling for village health workers in rural Qinghai, China

**DOI:** 10.1371/journal.pone.0324372

**Published:** 2025-06-26

**Authors:** Qiong Wu, Xinya Zhu, Tao Xu, Xiaotong Wang, Na Meng, Yanfeng Zhang, Suying Chang

**Affiliations:** 1 Department of Integrated Early Childhood Development, Capital Institute of Pediatrics, Beijing, China; 2 Child Health and Development Section, UNICEF China, Beijing, China; 3 Child and Adolescent Health Care Department, National Center for Women and Children’s Health, National Health Commission of the PRC, Beijing, China; Mizan-Tepi University, ETHIOPIA

## Abstract

**Background:**

Counselling is an effective behavior change intervention for improving infant and young child feeding (IYCF). The Maternal and Health Department of National Health Commission China and United Nations Children’s Fund (UNICEF) China Office jointly developed a combined online and in-person IYCF counselling training course for primary health workers in rural China. This study aims to test feasibility of the combined training model in rural China.

**Methods:**

The study was conducted between September 2021 and February 2022 in five counties in Qinghai Province, China. We implemented a “2+1” training model for village health workers in the five counties. This model consisted of a 2-day online training session followed by a 1-day in-person training session. After completing the training, each village health workers were requested to provide 12 IYCF counselling services to caregivers of children aged 6–23 months in their villages. We assessed the IYCF knowledge score of village health workers before and after online training, and collected satisfaction level of village health workers and caregivers by using a scale of 1–5. Values ≥4 were considered a high degree of satisfaction. Furthermore, we presented the per-person cost of the “2+1” training model.

**Results:**

There were a total of 861 village health workers in the five counties, with 802 (93.1%) completed the 2-day online training course and 838 (97.3%) completed the 1-day in-person training course. The mean score of village health workers on IYCF knowledge significantly improved after 2-day online training (72.14 ± 17.84 before training vs. 82.00 ± 17.87 after training, P < 0.001), and their average satisfaction scores on the training were more than 4.5 for all items. Furthermore, more than 95% surveyed caregivers expressed satisfaction with the counselling services provided by village health workers, and around 80% caregivers reported that the counselling solved their feeding problems. The cost of “2+1” training model was ¥238.72 (US$33.38) per person.

**Conclusions:**

This study showed that the combined online and in-person IYCF counselling training is acceptable and feasible with lower cost in rural of Qinghai, which can be scaled up in other rural areas in China.

## Introduction

Nutritional status in the first few years of life is a key determinant of children’s health and, hence, the health of a country’s future adult population [1]. Appropriate infant and young child feeding (IYCF) is the foundation of good nutritional intake, and promoting appropriate IYCF practices makes an important contribution to child survival, growth, and development [2–4]. However, the IYCF practices were not optimal in China. The national exclusive breastfeeding rate under six months was only 34.1% in 2017 [5]; and the prevalence of minimum dietary diversity (MDD), minimum meal frequency (MMF) and minimum acceptable diet (MAD) among children aged 6–23 months in rural areas in 2016–2017 was 60.6%, 72.4%, and 43.4%, respectively [6].

Evidence has shown that counselling is an effective behavior change intervention for improving breastfeeding and complementary feeding [7–8], and high-quality and timely IYCF counselling is also an effective strategy to improve the nutritional status of children [9–10]. Although China has been implementing a national program “Basic Public Health Service” since 2009, in which health workers are asked to provide face-to-face guidance on IYCF to pregnant women and mothers throughout antenatal and postnatal care [11], studies in some rural areas showed that only one-fourth mothers reported they received feeding information from health facilities [12]. Moreover, another national program called “Nutrition Improvement among Children in Poor Rural Regions Programme” has been implementing since 2012, which provide soybean powder-based complementary food supplement called YingYangBao (YYB) to 6–23 months children in rural areas daily for free, and the program had covered more than 832 counties by 2019 in China [13]. However, services of IYCF education were not emphasized in the programme, and gaps still exist as there are inadequate health care awareness, feeding knowledge and skills among caregivers in rural China. A recent study conducted in Liangshan in 2018 showed that less than 50% caregivers knowing key information related to child nutrition [14], which indicated that the gaps exist between health workers and caregivers when disseminating health information. Therefore, much effort is required to strengthen the quality of IYCF counselling for township and village health workers in China.

In 2019, the National Health Commission (NHC) of China and United Nations Children’s Fund (UNICEF) China jointly adapted “The Community-based Infant and Young Child Feeding Counselling Package” [15] to the local context, developed the IYCF counselling cards and published a book titled *Infant and Young Child Feeding (IYCF) Counselling: A Training Curriculum and Practical Guide for Primary Health Workers* (“IYCF counselling package” for short) in Chinese in July of 2021 [16]. The training package adopted interactive and participatory learning approaches to equip primary health workers with relevant updated knowledge and counselling skills on the recommended IYCF practices for children under 2 years aligned with both national and international IYCF guidelines through the 3-day face-to-face training. The package also included a set of 16 IYCF counselling cards with well-designed images to facilitate health worker’s counselling [17]. Qinghai Province was selected to pilot the IYCF counselling training package first in China, which a 3-day in-person training of trainer (TOT) at provincial level was conducted in December 2019, and five 3-day in-person TOT at county level was conducted in five counties in October 2020.

As information technology continues to advance, online training programs have experienced rapid growth, allowing users to engage with the programs in a flexible manner and at their own pace [18,19]. Moreover, online training can access to a greater number of clinicians, especially in rural areas [19]. Available evidence indicates that online learning can be just as effective as traditional learning [18]. Furthermore, due to the COVID-19 outbreak, 3-day face-to-face training among village health workers became difficult. Therefore, based on the adapted IYCF counselling package, National Center for Women and Children’s Health, National Health Commission and UNICEF China Office jointly developed by a 2-day online IYCF counselling training course in August 2021, which can be easily accessed by more primary health workers in China. In addition, as a supplement to the online course, a 1-day in-person training course was developed to give village health workers the chance to practice what they has been learned online.

Given the persistent challenges in improving IYCF practices in rural China and the introduction of the novel “2+1” training model, there is an urgent need to explore its feasibility and acceptability among village health workers in Qinghai Province. This study aims to fill this knowledge gap by evaluating the practicality of this new training model in the local context, with the main goal of providing evidence-based policy recommendations to national and local government.

## Materials and methods

### Study design

The study was conducted between September 2021 and February 2022 in five counties in Qinghai Province, China. Village health workers and caregivers with children under 2 years old in the five counties were invited to participate this study.

At the beginning of the study, village health workers were required to complete a 2-day online training course by themselves. Subsequently, a total of 31 1-day in-person IYCF practice training sessions were conducted for village health workers in the five counties (“2+1” training model). After training, each trained village health workers were requested to provide 12 IYCF counselling services to children and their caregivers in their villages.

We carried out pre-test and post-test of IYCF knowledge of village health workers before and after the online training course. In addition, data on village health workers’ satisfaction on the training and caregivers’ satisfaction on IYCF counselling services provided by village health workers were also collected to assess the feasibility of the “2+1” training model for village health workers.

### Study setting

Qinghai Province lies in northwest China, with an area of around 720,000 km^2^ and an average altitude of 9,900 feet (3,000 meters). There are 34 counties in Qinghai Province and most of them are agricultural and pastoral areas. The Qinghai resident per capita disposable income in 2020 was ¥12,342 (US$1,939.19,) for rural people [20], which was far lower than the average national income (¥17,131; US$2,691.65) [21]. The IYCF counselling training program was carried out in Menyuan County, Gonghe County, Tongren County, Pingan County and Datong County. In total, there were 66 townships, 680 villages, 861 village health workers and 15,643 children under 2 years old in these five counties in 2021.

### “2+1” training modality

The online IYCF counselling training course was developed by UNICEF China and National Center of Women and Children’s health, China CDC, which was free and publicly available in Chinese at www.chinawch.org.cn. The contents of the online training course was based on the adapted IYCF counselling package [16,17]. There are a total of 15 chapters comprising 38 learning objectives covering topics on breastfeeding, complementary feeding, women nutrition, and counseling skills. Each learning objective has a duration ranging from 3 to 30 minutes, and it takes around 2 days to complete the whole course. Participants can arrange their study time flexibly.

The online training course is presented in the form of pre-recorded video training. In each video, one trainer and four trainees demonstrate the training content in a scenario-based way. During the process, various interactive teaching methods are employed, including group discussions, demonstrations, and exercises to convey IYCF knowledge as well as counselling skills.

At the beginning of the online training course, health workers has to fill out a pre-test questionnaire on IYCF knowledge at the website (www.chinawch.org.cn), and at the end of the course, they has to complete the same questionnaire. Both pre-test and post-test consist of 20 questions of IYCF knowledge, with each question carrying 5 scores, resulting in a total of 100 scores for all the questions. Once the course was completed and a passing score on the tests was achieved, a certificate of completion will be provided to every trained health workers.

As a supplement to online course, the 1-day in-person training session was developed to strengthen the health workers’ practical skills on IYCF counselling. This session covered a wide range of practical aspects, such as practices of different breastfeeding positions and attachment, how to design one-day complementary foods for different age-groups, counselling exercises, field practice and assessments. Interactive and participatory learning approaches were used when conducting the training.

In September 2021, village health workers in the five counties were first asked to self-study the 2-day online course at their own home. In Tongren County, most of village health workers were Tibetan and unable to understand mandarin in the online course; therefore, they were gathered to study the online course together by using one account and a county level trainer translated the content of the online course at the same time. In addition, 30 village health workers in Gonghe County did not have computers and studied the online course together by using one account as well.

After all the village health workers completed the online training course, 1-day in-person practice training sessions were carried out from October to December 2021 in the five counties. In each county, village health workers from two or three townships nearby were gathered and trained. To ensure better-quality training, around 30 participants with at least 2 county level trainer were required for each training session, and a total of 31 in-person training was conducted. The county level trainer were trained in 3-day in-person TOT in October 2020. In addition, the IYCF counselling cards were printed and distributed to all training participants.

### IYCF counselling services for caregivers

After “2+1” training, each trained village health worker was encouraged to provide IYCF counselling service for children during their routine childcare services and required to provide 12 IYCF counselling services for caregivers of children under 2 years old in their own villages in two months. During each counselling session, village health workers used counselling skills to ask a series of questions related to breastfeeding and complementary feeding, such as the frequency of feeding, the amount of food or milk given, the texture of complementary foods, and the types of food included in the child’s diet. Based on the caregivers’ responses, the health workers assessed and analyzed the feeding problems, then provide caregivers with advice. Each counselling session lasted about 10–30 minutes. They also were required to record the counselling details on a form. This form included child’s age (months), sex, main caregivers, and the feeding problems identified by them and the advice they provided.

### Satisfaction surveys

We conducted a village health workers’ satisfaction survey and caregivers’ satisfaction survey in February 2022, respectively. The online survey tool ‘Sojump’ was used to collect data for the surveys.

For the village health workers’ satisfaction survey, we aimed to understand their satisfaction on “2 + 1“ training course, including training facilities and equipment, time arrangement, teacher’s teaching level, training aid in the course, material for training, practice section, and training format (S1 File). There were a total of 38 items in the village health workers’ satisfaction questionnaire, and each of these items received a numerical score on a scale of 1–5, from extremely satisfied to extremely dissatisfied (1: Extremely dissatisfied; 2: Dissatisfied; 3: Neither satisfied or dissatisfied; 4: Satisfied; and 5: Extremely satisfied). We also asked the health workers to assess the difficulty level and duration of both 2-day online training and 1-day in-person training, and collect their characteristics information (such as sex, age, education and workload). We set up the questionnaire on the ‘Sojump’ platform and got the quick response code (QR) of the electronic questionnaire. All the trained health workers received the QR code through their WeChat and scanned the QR code to answer the questions online.

For the caregivers’ satisfaction survey, we conducted the data collection 2–8 weeks after the initial IYCF counselling session in February 2022. We developed the questionnaire to collected data on whether caregivers received IYCF counselling services provided by village health workers, whether caregivers were satisfied with health workers’ altitude, whether health workers used counselling card during the counselling, and whether the counselling solved caregivers’ feeding problems. We sent the QR code of the questionnaire to caregivers through the three-tier health care system (county-township-village), which was in lined with our previous paper [22]. We initially sent the QR code to the county coordinator, then the township coordinator and village health workers. Village health worker explained the study and sent the QR code to caregivers through WeChat. Caregivers scanned the QR code and filled in the questionnaire online.

### Sample size and sampling

For village health workers, we planned to train all of them in the five counties, and sample size was 861.

For caregivers in the caregivers’ satisfaction survey, the sample size of was based on the estimated prevalence of caregivers’ satisfaction on counselling (80%); with a 4.0% desired level of absolute precision, 5% significant level and 80% power, we calculated that a sample size of 818 caregivers of children aged 0–23 months would be sufficient. Considering a 20% non-response rate, we supposed to recruit 1023 caregivers in this survey, 200 caregivers in each county. Convenience sampling method was used to obtain the target sample size in each county.

### Outcomes

The primary outcome measure was the training coverage, which was defined as the proportion of village health workers completed the 2-day online training and the 1-day in-person training course respectively.

The secondary outcome measure was IYCF knowledge change of village health workers, which was assessed through pre-test and post-test of the 2-day online training course.

The third outcome measure was village health workers’ satisfaction on the training and caregivers’ satisfaction on IYCF counselling services provided by village health workers. Satisfaction level was assessed by using a scale of 1–5. The scores of 4 or higher were considered a high degree of satisfaction.

We also reported the main feeding problems identified by trained village health workers during counselling services.

Furthermore, we presented the total cost of the “2+1” training model, as well as the unit cost per person for the training. Since village health workers completed the 2-day online training at their own home, there were no costs incurred during this online phase. To calculate the overall cost, we thus gathered data on the expenses related to trainers, meal allowances, accommodation costs, venue rental, and material fees for the 1-day in-person training across the five counties, and summed up these costs.

### Statistical analysis

Questionnaire data were uploaded to the Sojump platform and we download the Microsoft Excel sheet for the analysis. We analyzed data with SAS 9.4 for Windows (SAS Institute, Cary, NC). Numbers and proportions were used to describe categorical variables, mean and standard deviation (SD) were used to describe continuous variables in normal distribution, and medians and interquartile ranges (IQRs) in non-normal distribution. To compare the scores of IYCF knowledge test before and after the 2-day online training, we used an independent samples t-test. We considered two-tailed P-values of <0.05 for a significant difference.

### Ethical approval

Ethical clearance was obtained from the Capital Institute of Pediatrics in Beijing(SHERLL2021041). Each WeChat questionnaire has an electronic informed consent and participating village health workers or caregivers read the informed consent and clicked “Agree to participate” before they answered the questions.

## Results

### Training coverage

There were a total of 861 village health workers in the five counties, with 802 (93.1%) completed the 2 day online training course and 838 (97.3%) completed the 1-day in-person training course (Table 1).

**Table 1:** Training coverage of 2-day online and 1-day in-person training courses.

	Menyuan County (N = 156) n (%)	Gonghe County (N = 198) n (%)	Tongren County (N = 98) n (%)	Datong County (N = 289) n (%)	Pingan County (N = 120) n (%)	Total (N = 861) n (%)
Proportion of village health workers completed the 2-day **online** training course	144 (92.3)	198 (100.0)	98 (100.0)	242 (83.7)	120 (100.0)	802 (93.1)
Proportion of village health workers completed the 1-day **in-person** training course	156 (100.0)	198 (100.0)	85 (86.7)	279 (96.5)	120 (100.0)	838 (97.3)

### Changes of IYCF knowledge before and after online training

A total of 690 village health workers completed the pre-test and post-test of online training course. Due to local language barriers, there were 82 village health workers in Tongren Countyand and 30 village health workers in Gonghe County, who lacked computers, studied the online training course together using one account. As a result, they did not complete the pre-test and post-test. Table 2 shows the mean IYCF knowledge score of village health workers significantly improved after online training (72.14 ± 17.84 before online training vs. 82.00 ± 17.87 after online training, P < 0.001).

**Table 2:** Mean scores of IYCF knowledge test before and after online training.

County	N	Scores before training (Mean±SD)	Scores after training (Mean±SD)	*P*-value
**Menyuan**	144	69.97 ± 16.49	80.00 ± 17.54	<0.001
**Gonghe**	168	79.14 ± 18.36	87.47 ± 15.23	<0.001
**Tongren**	16	83.13 ± 16.82	83.75 ± 18.21	0.927
**Datong**	242	67.4 ± 16.21	78.86 + 17.36	<0.001
**Pingan**	120	72.94 ± 18.36	82.82 ± 20.74	<0.001
**Total**	690	72.14 ± 17.84	82.00 ± 17.87	<0.001

### Village health workers’ basic information and satisfaction on the training course

A total of 773 trained village health workers in five counties completed the village health workers’ satisfaction survey. The median age of village health workers in five counties was around 45 years old (Table 3). In Menyuan County and Tongren County, around 60% trained village health workers were male, while there are more female village health workers than male in other three counties.

**Table 3:** Characteristics of village health workers in five counties.

	Menyuan County (N = 174)	Gonghe County (N = 146)	Tongren County (N = 78)	Datong County (N = 264)	Pingan County (N = 111)	Total (N = 773)
	n (%)	n (%)	n (%)	n (%)	n (%)	n (%)
**Age in years,Median (Q1,Q3)**	43.5 (37,50)	40 (29,48)	39 (31.5,49)	48 (41,53)	46 (38,52)	45 (35,51)
**Sex**						
Male	113 (64.9)	49 (33.6)	45 (57.7)	96 (36.4)	17 (15.3)	320 (41.4)
Female	61 (35.1)	97 (66.4)	33 (42.3)	168 (63.6)	94 (84.7)	453 (58.6)
**Education background**						
**Basic education**						
Primary school	7 (4.0)	6 (4.1)	11 (14.1)	5 (1.9)	12 (10.8)	41 (5.3)
Middle school	122 (70.1)	82 (56.2)	40 (51.3)	145 (54.9)	61 (55.0)	450 (58.2)
High school	45 (25.9)	58 (39.7)	27 (34.6)	114 (43.2)	38 (34.2)	282 (36.5)
**Professional education**						
Professional high school	18 (10.4)	6 (4.1)	8 (10.3)	69 (26.1)	43 (38.8)	144 (18.6)
Technical secondary school	118 (67.8)	78 (53.4)	41 (52.5)	81 (30.7)	36 (32.4)	354 (45.8)
Junior college	34 (19.5)	46 (31.5)	23 (29.5)	98 (37.1)	28 (25.2)	229 (29.6)
University	4 (2.3)	16 (11.0)	6 (7.7)	16 (6.1)	4 (3.6)	46 (6.0)
**Number of children under 2 years old served last month, Median(Q1,Q3)**	6 (3,15)	8 (3,18)	8 (4,18)	8 (4,16)	5 (2,10)	7 (3,15)

More than 90% village health workers in five counties attended middle school or above, while the professional education of village health workers varied. The child health care workload of village health workers averaged around 7 children under 2 years old last month in five counties.

**Fig 1:**
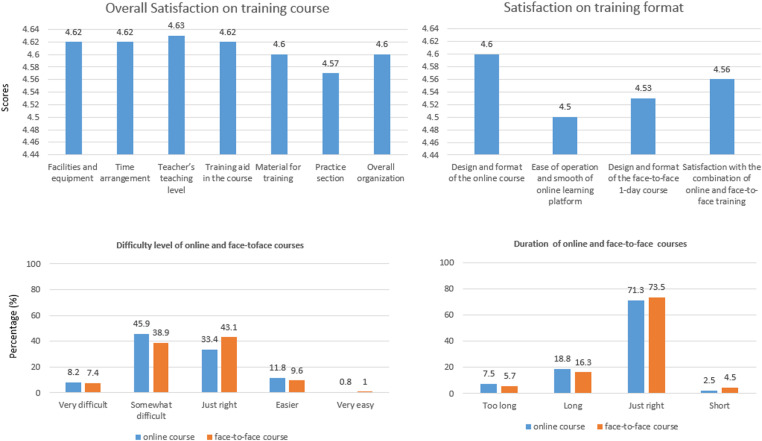
Village health workers’ satisfaction on the training course.

The average satisfaction scores for all items exceeded 4.5 (see Fig 1). Around 80% village health workers in five counties thought that the difficulty level of online training course was just right or somewhat difficult. Similarly, 82% of them considered the one-day in-person training to be at a just right or somewhat difficult level. Furthermore, 71.3% and 73.5% village health workers thought the duration of online courses and one-day in-person training were appropriate, respectively.

### Main feeding problems identified by trained village health workers during counselling services

All 838 trained village health workers reported completing 12 counselling session each. Based on this, the estimated total number of children counselled was approximately 10,000, However, in the five counties, only 4,815 counselling services were properly recorded and submitted as requested. In the recorded counselling, 20.0% were children under 6 months, 29.3% were children aged 6–11 months, and 50.8% were children aged 12–23 months. The gender ration is 1.06.

Table 4 shows the breastfeeding and complementary feeding problems identified by village health workers during the counselling services. There were 1,606 breastfeeding problems were identified, with mothers’ perceived insufficient milk supply, incorrect breastfeeding position, and weaning before 2 years old ranking the top 3.

**Table 4:** Feeding problems identified by village health workers.

Feeding problems identified	No. of problems	Percentage (%)
**Breastfeeding problems**	**1606**	
Self-confessed lack of breast milk	396	24.7
Incorrect breastfeeding position	365	22.7
Weaning before 2 years old	245	15.3
Poor attachment	207	12.9
Sore and cracked nipples	117	7.3
Not exclusively breastfeeding under 6 months of age	79	4.9
Others	197	12.3
**Complementary feeding problems**	**3906**	
Dietary diversity needs to be improved	1179	30.2
Picky eating	537	13.7
Dislike YingYangBao	334	8.6
Disease related complementary feeding	320	8.2
Did not feed meat	295	7.6
Did not feed beans	175	4.5
Insufficient amount of complementary food	141	3.6
Others	925	23.7

There were a total of 3,906 complementary feeding problems identified, among which 30.2% were that children’s dietary diversity needs to be improved and 13.7% were picky eating.

### Caregivers’ satisfaction on IYCF counselling services

A total of 1,106 caregivers who received IYCF counselling services were surveyed on the satisfaction in the five counties. More than 95% caregivers were satisfied with the counselling services. Around 80% caregivers reported that the counselling absolutely solved their feeding problems. More than 90% village health workers used counselling card when they provided IYCF counselling services.

### Cost of “2+1” training

Table 5 shows the cost for “2+1” training. The cost of “2+1” training village level IYCF training was ¥238.72 (US$33.38) for training per person.

**Table 5:** The costs of “2+1” training method.

Items of money spent actually on	“2+1” training for village health workers in 2021
Number trained	838
No. of training session	31
Cost for trainers	82900
Meal fee	58660
Accommodation expenses	16260
Venue hire	0
Material fee	42229
Total (¥/$)	200,049/28,007
Unit Cost for training per person (¥/$)	239/33

## Discussion

This study demonstrated that the combined “2+1” training model is both feasible and highly acceptable for primary health workers in Qinghai, China. The training coverage exceeded 80% for both 2-day online training and 1-day in-person training. Village health workers exhibited significantly improvement in their IYCF knowledge after the online training, and expressed high satisfaction with both online and in person training. Moreover, over 95% caregivers were satisfied with the IYCF counselling services provided by their village health workers. The cost per person for the “2+1” training for village health workers was was ¥239 (US$33).

These findings indicated the “2+1” training model for village heath workers was acceptable, feasible and desirable in rural China. Particularly, the relatively low costs of the combined training method provide the possibility of nationwide scale-up of the training. Online training enables greater time flexibility and geographic accessibility [18], which is friendly to primary health workers who has heavy workload and live in remote areas. It is also important to integrate 1-day in-person practice training course to strengthen health workers’ knowledge and skills learned from the online training course. Most people who received the training thought the 1-day in-person training session was very practical and interactive, and it inspired their motivation and enthusiasm for providing IYCF support.

However, some challenges need to be addressed when conducting online IYCF training in rural China. First, language difficulties should be considered for some ethnic minorities, as they cannot understand mandarin. In our study, most of village health workers in Tongren County were Tibetan, and they were not able to understand mandarin in the online course; therefore only 16 village health workers completed the online training course, suggesting that local language translation of the training course is needed. Another challenge was short of computer in some remote areas. In our study, there were 30 village health workers in Gonghe County did not have computers, and they watched the online courses together by using the same account. With the widespread use of smartphones in China [23], learning online course through smartphones should be considered. Thus, after this pilot, we added a IYCF counselling training module into the Family Health App used by many primary health workers in China to support their learning through smartphone.

It was helpful to ensure that tools, surveys, and records are available in both electronic version and paper version, and in a simple format. The study showed that the IYCF counselling cards are a valuable tool for primary health workers, particularly in rural areas, and should be promoted and widely distributed. The counseling card could better assist village health workers during the counseling services, such as guiding the development of topics, establish trust with parents, and provide practical feeding suggestions to parents. Additionally, counselling service records and other assessment forms should be simplified where possible, preferably using electronic formats.

Our results also proved that China’s rural three-tier healthcare system (county-township-village) is a good approach for the cascade IYCF training for health workers. In this study the IYCF training was well organized and implemented successfully in Qinghai China using three-tier health care system. Before the village level training, we conducted a 3-day face-to-face training of trainer (TOT) at provincial level in December 2019, and five 3-day face-to-face TOTs at county level in October 2020, ensuring enough qualified trainers for village level training. Each training session was equipped at least two trainers including one from county level and the others from township level. The three-tier healthcare system also supported good programme monitoring and supervision works. The study showed that it was feasible to use a variety of training modalities suited to the different levels, time availability and experiences of the participants. This also mitigated limitations related to the COVID-19 pandemic.

This study also analyzed the identified feeding problems during providing IYCF counselling services, most of which are covered by our training courses. Village health workers identified that perceived insufficient milk supply (PIMS) was the most common breastfeeding problem (24.7%), which was in line with the previous systematic review [24]. Evidence has shown that PIMS is one of the main reasons given for formula supplementation and breastfeeding discontinuation [24]. In addition, proper positioning and good attachment play a significant role in initiating and sustaining exclusive breastfeeding. However, incorrect breastfeeding position and poor attachment were still common among caregivers. For complementary feeding problems, poor dietary diversity was most common among children aged 6–23 months. In our training course we made a detail explanation on how to solve these problems, so that village health workers could provide appropriate advice for caregivers. In our study, around 80% caregivers reported that the counselling absolutely solved their feeding problems, which indicate that the training was productive.

Our study also has some limitations. First, we did not conduct the health facility survey to assess IYCF counselling service quality of village health worker. Second, we only conducted this study in five counties in rural Qinghai, China, so the generalization of the results requires caution. Third, given the feasibility study design, we did not evaluate the effectiveness of “2+1” training model on IYCF outcomes. Future studies need to incorporate pre-post assessment or randomized control trial to rigorously evaluate the impact of the”2 + 1”training model on IYCF practices and health outcomes.

## Conclusions

This feasibility study showed that the combined 2-day online and 1-day in-person IYCF counselling training for primary health workers in rural China is acceptable, feasible and desirable. The training model can be extended to other rural areas in China. This successful feasibility study laid the foundation for further studies need to be conducted to evaluate the effectiveness of the combined training, and then the training model can be extended to other rural China.

## Supporting information

S1 File IYCF training satisfaction questionnaire for village village health workers. (DOCX)

S1 Dataset Dataset for village health workers’ satisfaction. (XLSX)

S2 Dataset Dataset for counselling. (XLSX)

S3 Dataset Dataset for caregivers’ satisfaction. (XLSX)

## Abbreviations

IYCF: Infant and young child feeding
TOT: training of trainer.
