# Supplementary material for: Feasibility of a combined online and in-person training model on Infant and Young Child Feeding (IYCF) counselling for village health workers in rural Qinghai, China
Source: PLoS One. 2025 Jun 26;20(6):e0324372. doi: 10.1371/journal.pone.0324372 (PMC12200772; doi:10.1371/journal.pone.0324372)
Supplement: S1 File — (DOCX) [file pone.0324372.s001.docx]

**Supplementary file 1**

**IYCF training satisfaction questionnaire for village village health workers**

1. Basic Information (ID) :

1. Your county/township/village

2. Your name

3. Your gender 1.male 2.female

4. Your age (1 year old)

5. Your contact information (mobile)

6. Your level of literacy

6.1 Basic Education degree: 1. Primary School 2. Junior High School 3. Senior High School

grade: _____Grade

6.2 Vocational education qualification: 1. Vocational highschool 2. technical secondary school 3. Junior College

6.3 College completion or not: 1. Yes 2. No

7. Have you had any previous training experience in online courses? 1. Yes 2. No

8. The total number of children under the age of 2 in your village:

9. How many days a week you work in the village health office: days

10. How many days a week you provide health services to children: days

11. The total number of people who came to the health service here last month:

12. How many of these patients were children aged 0-2?

13. Of these children, how many are there in each of the following?

13.1 Outpatients ________________

13.2 Health checkups ________________

13.3 Other ________________

2. Overall evaluation of the training courses:

(1) Your satisfaction with the training hardware conditions such as room, seating arrangement, stationery, sanitation, etc

1: Extremely satisfied; 2: Satisfied ; 3: Neither satisfied or dissatisfied; 4: Dissatisfied; and 5: Extremely dissatisfied

(2). Your satisfaction with the schedule of the course

1: Extremely satisfied; 2: Satisfied ; 3: Neither satisfied or dissatisfied; 4: Dissatisfied; and 5: Extremely dissatisfied

If you fill in 1,2, jump to question 3, if you fill in 3,4,5, jump to (2)

(2.1) If you are not satisfied with the schedule, which of the following conditions is true

1. Course schedule is too tight 2. Course schedule is too loose 3. Other reasons (fill in)

(3). Your satisfaction with the teacher's teaching level

1: Extremely satisfied; 2: Satisfied ; 3: Neither satisfied or dissatisfied; 4: Dissatisfied; and 5: Extremely dissatisfied

(4). Your satisfaction with the teaching aids used by the teachers in the training

1: Extremely satisfied; 2: Satisfied ; 3: Neither satisfied or dissatisfied; 4: Dissatisfied; and 5: Extremely dissatisfied

(5). Your satisfaction with the information used in the training, such as student materials, exercise forms and consultation cards

1: Extremely satisfied; 2: Satisfied ; 3: Neither satisfied or dissatisfied; 4: Dissatisfied; and 5: Extremely dissatisfied

(6). Your satisfaction with the practical activities and internships used in the training

1: Extremely satisfied; 2: Satisfied ; 3: Neither satisfied or dissatisfied; 4: Dissatisfied; and 5: Extremely dissatisfied

(7). Your satisfaction with the overall organization of the training course

1: Extremely satisfied; 2: Satisfied ; 3: Neither satisfied or dissatisfied; 4: Dissatisfied; and 5: Extremely dissatisfied

1. Evaluation of the training course content
2. (1)Do you think the training content is helpful to your daily work?

1. Very useful 2. useful 3. General 4. Basically useless 5.No use at all

(2)If you choose 1,2,3, jump to question (2), if you choose 4,5, jump to question 3

What topics do you think of the most useful training content for work (optional)

1. Breastfeeding 2. complementary food 3. counseling skills and tools 4. compliant feeding 5. clean hygiene 6. Other can be added (fill in)

(3) Satisfaction with the practicability and operability of the training content

1. Very satisfied 2. satisfied 3. General 4. not satisfied 5

(4) Overall satisfaction with the knowledge, ideas, skills and other contents learned in the training

1: Extremely satisfied; 2: Satisfied ; 3: Neither satisfied or dissatisfied; 4: Dissatisfied; and 5: Extremely dissatisfied

1. Evaluation of the training form (this training is the first online video course learning, and then under 1 antenna)

Evaluation of online video courses:

(1) The degree of satisfaction of the design and form of the online course

1: Extremely satisfied; 2: Satisfied ; 3: Neither satisfied or dissatisfied; 4: Dissatisfied; and 5: Extremely dissatisfied

1. The difficulty level of the online course

1.Very difficult 2. Somewhat difficult 3. Just right 4. Easy 5.very easy

1. The duration of the online course study

1. Too long 2. long 3. Just right 4. short 5. Too short

(4) Online learning platform is easy to operate and smooth

1: Extremely satisfied; 2: Satisfied ; 3: Neither satisfied or dissatisfied; 4: Dissatisfied; and 5: Extremely dissatisfied

(5) The mastery of the content of this online course is compared with other online courses

1. Better mastery 2. Almost 3.Not well mastered

1. Satisfaction with the design and form of the offline 1-day course

1: Extremely satisfied; 2: Satisfied ; 3: Neither satisfied or dissatisfied; 4: Dissatisfied; and 5: Extremely dissatisfied

1. The difficulty degree of the one-day offline course
2. Very difficult 2.Somewhat difficult 3. Just right 4. Easy 5.very easy
3. One-day offline course learning duration

1. Too long 2. long 3. Just right 4. short 5. Too short

1. The mastery of the content of this offline course is compared with other offline courses

1. Better mastery 2. Almost 3.Not well mastered

1. Do you think the combination of online and offline training will help you?

1. Very helpful 2. Quite helpful 3. Moderately helpful 4. Not very helpful 5. Not helpful at all

1. The satisfaction degree of the combined online and offline training form

1: Extremely satisfied; 2: Satisfied ; 3: Neither satisfied or dissatisfied; 4: Dissatisfied; and 5: Extremely dissatisfied

1. Train trainees in self-assessment
2. The training results are inline with your intended goals

1: Extremely satisfied; 2: Satisfied ; 3: Neither satisfied or dissatisfied; 4: Dissatisfied; and 5: Extremely dissatisfied

1. The training stimulated your enthusiasm, enthusiasm and interest in infant feeding work.

1: Extremely satisfied; 2: Satisfied ; 3: Neither satisfied or dissatisfied; 4: Dissatisfied; and 5: Extremely dissatisfied

1. Is this training enlightening for your work?

1: Extremely satisfied; 2: Satisfied ; 3: Neither satisfied or dissatisfied; 4: Dissatisfied; and 5: Extremely dissatisfied

1. How much can this training better help you to carry out infant feeding counseling?

1: Extremely satisfied; 2: Satisfied ; 3: Neither satisfied or dissatisfied; 4: Dissatisfied; and 5: Extremely dissatisfied

1. After this training, are you willing to carry out more infant feeding consultation work?

1: very willing 2:It depends on the time. I'm not sure. 3:not willing
